# Comparative proteomic analysis of pituitary glands from Huoyan geese between pre-laying and laying periods using an iTRAQ-based approach

**DOI:** 10.1371/journal.pone.0185253

**Published:** 2017-09-25

**Authors:** Xinhong Luan, Zhongzan Cao, Zhe Xing, Mei Liu, Ming Gao, Bo Meng, Ruiming Fan

**Affiliations:** Key Laboratory of Zoonosis of Liaoning Province, College of Animal Science & Veterinary Medicine, Shenyang Agricultural University, Shenyang, P.R. China; Universite de Rouen, FRANCE

## Abstract

In this study, we performed a comprehensive evaluation of the proteomic profile of the pituitary gland of the Huoyan goose during the laying period compared to the pre-laying period using an iTRAQ-based approach. Protein samples were prepared from pituitary gland tissues of nine pre-laying period and nine laying period geese. Then the protein samples from three randomly selected geese within each period were pooled in equal amounts to generate one biological sample pool. We identified 684 differentially expressed proteins, including 418 up-regulated and 266 down-regulated proteins. GO annotation and KEGG pathway analyses of these proteins were conducted. Some of these proteins were found to be associated with hormone and neurotransmitter secretion and transport, neuropeptide signalling and GnRH signalling pathways, among others. Subsequently, the modification of the abundance of three proteins (prolactin, chromogranin-A and ITPR3) was verified using western blotting. Our results will provide a new source for mining genes and gene products related to the egg-laying performance of Huoyan geese, and may provide important information for the conservation and utilization of local goose breeds.

## Introduction

The Liaoning Huoyan goose is considered a national treasure by the Chinese goose industry and was listed as one of the nationally protected domestic animals by the Chinese government (http://www.moa.gov.cn/zwllm/tzgg/gg/201402/t20140220_3791641.htm). It is famous for its high laying performance domestically and on a global scale. However, problems associated with variety degeneration have emerged recently, with decreases in egg production being very prominent, which hinder goose industry development. Investigating the molecular mechanisms underlying the reproductive biology and improving the laying performance of Huoyan geese is important for the conservation and utilization of this famous local goose breed.

The hypothalamic-pituitary-gonadal (HPG) axis is critical in the development and regulation of the reproductive and endocrine systems in poultry. The neuropeptides and hormones secreted from the HPG are key regulators of this significant axis pathway and play pivotal roles in the control of poultry reproduction [1]. Several studies have identified genes associated with reproductive traits in the HPG of birds using genomic and transcriptome approaches, such as simple gene investigation, high-throughput expressed sequence tag analysis, suppression subtractive hybridization, and RNA-Sequencing [1–8]. Selecting for an individual gene in birds is an effective alternative for improving economically important traits, but this approach may not guarantee success if multiple factors and their dynamic interactions are involved in the phenotype expression of the target trait [9]. Proteomics offers a new platform for studies of complex biological functions involving large numbers and networks of proteins [10] and allows us to obtain substantial information regarding protein posttranslational modification, proteolytic processing, and mRNA alternative splicing that could not be achieved by DNA sequence analysis alone [11]. Many proteomic platforms have been developed for the qualitative and quantitative characterization of protein mixtures and post-translational modifications, such as 2-DE [12], LC-MS/MS [13]. To date, several research groups have reported the application of conventional proteomics approaches in the study of bird reproduction. For determining molecular markers associated with egg production, 2-DE has been used to identify hypothalamic proteins associated with high egg production in domestic chickens [14]. This approach has also been applied to analyse the serum protein composition and construct a proteome reference map for single-comb White Leghorn hens at different developmental stages [15]. In addition, Nam Soo Kim *et al*. used the 2-DE method to show that anterior gradient-2 (AGR-2) may be an oviduct-specific protein involved in egg formation and epithelial cell differentiation during the egg-laying period of hens [16]. In general, 2-DE provides a visual representation of the proteome in which distinct protein isoforms resulting from the changes in Mr and/or p*I* can be observed. However, 2-DE suffers from low throughput non-quantitative information coupled with difficulties in separating and/or detecting low abundance proteins, post-translationally modified proteins [17, 18], and proteins with a p*I* value lower than 4 or higher than 9 [19]. Recently, a new technique known as iTRAQ (isobaric tags for relative and absolute quantitation) followed by nano liquid chromatography-mass spectrometry (Nano LC-MS/MS) has been applied for proteomic quantitation. This method overcomes some of the limitations of other conventional proteomics techniques and improves the throughput of proteomic studies. This is one of the most highly sensitive proteomic technologies because it can detect and quantitatively analyse low-abundance proteins in complex biological samples [20].

The activation and maintenance of normal follicular functions are dependent on gonadotropins secreted by the pituitary, which in turn is regulated by the hypothalamic gonadotropin releasing hormone (GnRH) [14]. Minute differences in hypothalamic or pituitary functions, might affect reproductive processes, including folliculogenesis, ovulation, oviposition, and incubation behaviour [21]. Consequently, the pituitary gland is an ideal tissue to study to elucidate the molecular mechanisms associated with egg production. Information about the protein profiles in the pituitary at different reproductive periods is essential for studying the molecular genetic mechanisms of egg-laying. In this study, we applied iTRAQ integrated with liquid chromatography-tandem mass spectrometry (LC-MS/MS) analysis to identify differentially expressed proteins in pituitary glands from Huoyan geese between the pre-laying period and laying period. The possible biological significance of these differentially expressed proteins was further evaluated using various bioinformatics programs. Several differentially expressed proteins were then validated using western blotting.

## Materials and methods

### Ethics statement

Experimental procedures were approved by the animal welfare committee of the College of Animal Science and Veterinary Medicine of Shenyang Agricultural University (No. 2013025) and performed in accordance with the Regulations for the Administration of Affairs Concerning Experimental Animals (China, 1988) and EU Directive 2010/63/EU for animal experiments. All of the surgeries were performed according to recommendations proposed by the European Commission (1997), and all efforts were made to minimize the suffering of the animals.

### Animal and tissue collection

The Huoyan geese were selected from the Liaoning Huoyan Goose Stock Breeding Farm and raised according to the farm program. During the experiment, geese were fed ad libitum with rice grain and supplemented with green grass or water plants whenever possible. Huoyan geese become sexually mature at approximately 7 months of age and reach the peak egg-laying stage in the following year. In the current study, goslings were purchased in the fall of the year and became sexually mature during the summer of the following year. Nine pre-laying period geese were killed by exsanguination at 6 months of age. Nine laying period geese were killed by exsanguination at 12 months of age. The pituitary gland tissues were quickly dissected, frozen in liquid nitrogen, and stored at −80°C until protein was prepared.

### Protein preparation

All pituitary tissues were homogenized in 0.5 ml STD lysis buffer (4% SDS, 150 mM Tris–HCl, 1 mM DTT, pH 8.0, and protease inhibitor). Then, the samples were sonicated and boiled at 100°C for 15 min followed by centrifugation at 14000 × g for 45 min at 25°C. The supernatant was precipitated overnight with cold acetone. After discarding acetone, air-drying, and the resulting pellet was dissolved in 30 μl STD buffer. Protein concentrations were determined using the BCA protein assay reagent (Beyotime Institute of Biotechnology, Shanghai, China). Protein samples were stored at −80°C until needed.

### Protein digestion and iTRAQ labelling

Protein digestion was performed according to the FASP procedure described by Wiśniewski et al. (2009) [22]. To reduce overall variability by minimizing individual heterogeneity [23], the protein samples from three randomly selected geese within each group were pooled in equal amounts to generate one biological sample pool, and there were three biological protein pools each in the pre-laying group and laying group. Protein (300 μg) from three equally pooled biological replicates was diluted with 200 μl UA buffer (8 M Urea, and 150 mM Tris-HCl, pH8.0) and loaded onto an ultrafiltration filter (30 kDa cut-off, Sartorius, Germany). Samples were centrifuged at 14,000×g for 15 min; 200 μl UA buffer was then added, followed by centrifugation for an additional 15 min. After discarding the supernatant, 100 μl 50 mM iodoacetamide in UA buffer was subsequently added to the filter, with oscillation at 600 rpm for 1 min. The samples were incubated for 30 min in darkness and then centrifuged at 14,000 × g for 10 min. The filters were washed twice with 100 μl UA buffer, and 100 μl dissolution buffer (50 mM triethylammonium bicarbonate at pH 8.5) was added to the filters, followed by centrifugation for 10 min. This step was repeated twice, and 40 μl trypsin buffer (2 μg trypsin in 40 μl dissolution buffer) was then added to each filter. The samples were oscillated at 600 rpm for 1 min and incubated at 37°C for 16–18 h. Finally, the filter unit was transferred to a new tube and centrifuged at 14,000 × g for 10 min. The resulting peptides were collected as a filtrate, and the peptide concentration was analysed at OD_280_ [22].

The resulting peptide mixture was labelled using the 8-plex iTRAQ reagent according to the manufacturer's instructions (Applied Biosystems). Three samples from the pre-laying group were labelled with mass 113, 116 and 117 isobaric iTRAQ tags, while the other three samples from the laying group were labelled with mass 114, 115 and 118 isobaric iTRAQ tags. The labelling solution reaction was then incubated at room temperature for 1 h prior to further analysis.

### Peptide fractionation with strong cation exchange (SCX) chromatography

The iTRAQ-labelled peptides were subjected to SCX fractionation in an AKTA Purifier 100 (GE Healthcare). The dried peptide mixture was reconstituted and acidified with 2 ml buffer A (10 mM KH_2_PO_4_ in 25% of ACN, pH 3.0) and loaded onto a polysulfoethyl (PolyLC Inc., Maryland, USA.) column (4.6 mm × 100 mm, 5 μm, 200 Å). The peptides were eluted at a flow rate of 1 ml/min with a gradient of 100% Buffer A for 25 min, 0–10% Buffer B (500 mM KCl, 10 mM KH_2_PO_4_ in 25% of ACN, pH 3.0) for 7 min, 10–20% Buffer B for 10 min, 20–45% Buffer B for 5 min, 45–100% Buffer B for 5 min, 100% Buffer B for 8 min, and finally 100% Buffer A for 15 min. The elution process was monitored by absorbance at 214 nm, and fractions were collected every 1 min. The collected fractions (approximately 30) were finally combined into 6 pools and desalted on C18 cartridges (Sigma, Steinheim, Germany). Each fraction was concentrated via vacuum centrifugation and reconstituted in 40 μl of 0.1% (v/v) trifluoroacetic acid. All samples were stored at -80°C until LC-MS/MS analysis.

### Liquid chromatography-tandem mass spectrometry (LC-MS/MS)

The iTRAQ-labelled samples were analysed using an Easy-nLC nanoflow HPLC system connected to a Q-Exactive mass spectrometer (Thermo Fisher, San Jose, CA, USA). A total of 5 μg of each sample was loaded onto a Thermo Scientific EASY column (2 cm × 100 μm, 5 μm-C18) using an auto sampler at a flow rate of 250 nl/min. The peptides were separated on a Thermo Scientific EASY column (100 mm × 75 μm, 3 μm-C18) using a segmented 2-h gradient from Solvent A (0.1% formic acid in water) to 35% Solvent B (84% acetonitrile in 0.1% formic acid) for 100 min, followed by 35–100% Solvent B for 8 min and then 100% Solvent B for 12 min. The column was re-equilibrated to its initial highly aqueous solvent composition before each analysis.

The peptides were subjected to the Q-Exactive mass spectrometer. MS data were acquired using a data-dependent top 10 method dynamically choosing the most abundant precursor ions from the survey scan (300–1800 m/z) for subsequent high-energy collisional dissociation (HCD) fragmentation in the positive ion mode. Determination of the target value is based on predictive automatic gain control (pAGC). Dynamic exclusion was used with a 40.0-s duration. The resolving power of the MS scan and the MS/MS scan at 200 m/z were set at 70,000 and 17,500, respectively. The top 10 most intense signals in the acquired MS spectra were selected for further MS/MS analysis. The isolation window was 2 m/z, the normalized collision energy was 30 eV, and the underfill ratio, which specifies the minimum percentage of the target value likely to be reached at maximum fill time, was defined as 0.1%. The maximum ion injection times were set at 10 ms for the survey scan and 60 ms for the MS/MS scans, and the automatic gain control target values for both scan modes were set to 3.0×10^−6^. The instrument was run with peptide recognition mode enabled.

### Sequence database searching and data analysis

The MASCOT 2.2 (Matrix Science, London, UK) and Proteome Discoverer 1.4 software (Thermo Scientific, San Jose, CA, USA) were used for identification and quantitative analysis. The raw files were analysed using the Proteome Discoverer software. Protein identifications were performed using the MASCOT search engine embedded into Proteome Discoverer, searching against the Uniprot database of Anatidae protein sequences (08-15-2014, 34815 entries, downloaded from: http://www.uniprot.org/). Search parameters were set as follows: monoisotopic mass, trypsin as the cleavage enzyme, two missed cleavages, peptide mass tolerance at ±20 ppm and MS/MS tolerance at 0.1 Da. Variable modifications were defined as oxidation of methionine and iTRAQ 8-plex labelled tyrosine, while lysine and the N-term of peptides labelled by iTRAQ 8-plex and carbamidomethylation on cysteine were specified as fixed modifications, with the Decoy database pattern = Reverse. The results were filtered based on the false discovery rate (FDR). All reported data were based on 99% confidence for protein identification as determined by FDR < 1%. FDR = N(decoy)*2/ ((N(decoy)+ N(target)) [24].

The relative quantitative analysis of the proteins in the samples based on the ratios of iTRAQ reporter ions from all unique peptides representing each protein was performed using Proteome Discoverer. The relative peak intensities of the iTRAQ reporter ions released in each of the MS/MS spectra were used. The summary of the intensities in all channels was employed as a reference for calculating the iTRAQ ratios of all reporter ions. The iTRAQ ratio of every group of proteins was obtained by using the intensity of each channel normalized to the reference [25]. For statistical analysis, Student’s *t*-test was employed to identify significant changes between the pre-laying group and laying group samples. The FDR value was further computed by using the *p*.*adjust* function in R (version 3.1.3, R Foundation for Statistical Computing, http://www.r-project.org). Proteins with a statistically significant iTRAQ ratio of > 1.2 or < 0.83 (FDR < 5%) were considered to be differentially expressed proteins (DEPs).

### Bioinformatics analysis

Sequence data of the selected the differentially expressed proteins were retrieved in batches in FASTA format from the UniProtKB database (Release 2015_03). The retrieved sequences were locally searched against the non-redundant protein database (NCBI nr) using the NCBI BLAST+ client software (ncbi-blast-2.2.28 + -win32.exe) to find homologous sequences from which the functional annotation was transferred to the studied sequences. In this study, the top 10 blast hits with an E-value of ≤ 1e - 3 for each query sequence were retrieved and loaded into Blast2GO (Version 2.8.0) for Gene Ontology (GO) mapping and annotation [26]. Unannotated sequences were then re-annotated with more permissive parameters. The sequences without BLAST hits and the unannotated sequences were then selected to go through InterProScan [27] against EBI databases to retrieve functional annotations of protein motifs and merge the InterProScan GO terms to the annotation set. The Gene Ontology (GO) mapping described the roles of proteins according to the following three domains: biological process (BP), molecular function (MF) and cellular component (CC) [28]. Following annotation and annotation augmentation steps, the studied proteins were blasted against KEGG genes (Birds) to retrieve their KEGG orthology identification and were subsequently mapped to pathways in the Kyoto Encyclopedia of Genes and Genomes (KEGG, http://www.genome.jp/kegg/) using the online KEGG Automatic Annotation Server (KAAS) (http://www.genome.jp/kegg/kaas/) [29, 30].

### Western blot analysis

Protein samples of pituitary tissues from the pre-laying and laying groups were extracted and identified using kits according to the manufacturer’s instructions (Applygen Co., LTD. Beijing, China). Equivalent amounts of total protein were subjected to 12% SDS-PAGE and then transferred to a nitrocellulose membrane. After blocking with 5% skim milk in PBS containing 0.1% Tween 20 (PBST) at 4°C overnight, the membranes were incubated separately with goat anti-prolactin antibody (sc-7805, Santa Cruz Biotechnology, USA), goat anti- chromogranin-A antibody (sc-23556, Santa Cruz Biotechnology, USA), and rabbit anti-ITPR3 (inositol 1,4,5-trisphosphate receptor, type 3) antibody (bs-6471R, Beijing Biosynthesis Biotechnology Co., LTD) overnight at 4°C. The membranes were subsequently incubated with donkey anti-goat IgG/HRP antibody (bs-0294D-HRP, Beijing Biosynthesis Biotechnology Co., LTD) or goat anti-rabbit IgG/HRP antibody (bs-0295G-HRP, Beijing Biosynthesis Biotechnology Co., LTD) for 1 h at 37°C. Finally, the bands were captured using a MicroChemi4.2 imaging system (DNR Bio-imaging Systems, Jerusalem, Israel), and densitometry analysis of protein bands was performed using the GelQuant software (DNR Bio-imaging Systems, Jerusalem, Israel). GAPDH (sc-20357, Santa Cruz Biotechnology, USA) was used as a reference protein to ensure equal loading. Triplicate experiments were performed for each sample. All data were analysed using SPSS 16.0 for Windows (SPSS Inc. Chicago, IL, USA). The data were analysed by Student’s *t*-test. The results are expressed as the mean ± SEM. *P* < 0.05 was considered to be statistically significant.

## Results

### Global profiling of proteins in pituitary gland tissues

**Fig 1:**
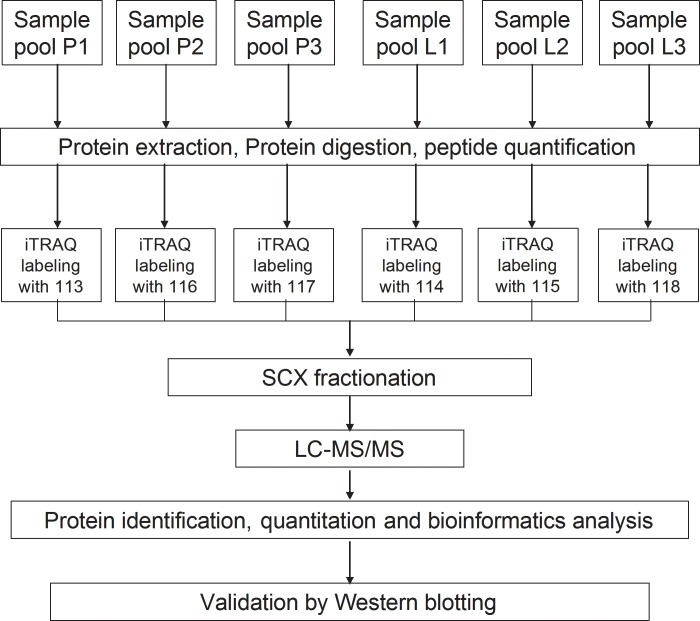
Experimental design and schematic diagram of the workflow used in this study.

**Fig 2:**
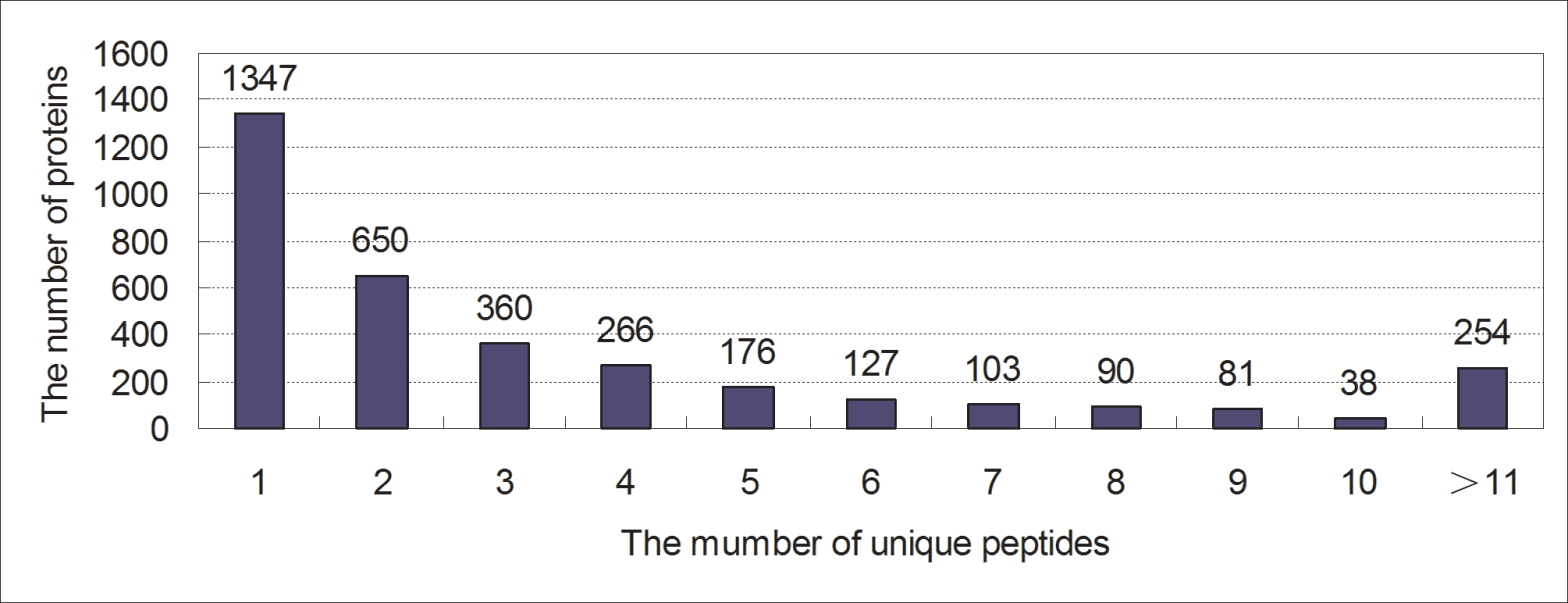
Unique peptides for the identified proteins. The number of proteins in each category is presented at the top of each bar.

We employed the iTRAQ labelling technology in combination with LC-MS/MS to investigate differentially expressed proteins in the pituitary of Huoyan geese between the pre-laying period and laying period. A schematic workflow illustrating the steps applied in this study is shown in Fig 1. A total of 3492 proteins were identified according to the standard of protein identification (one or more unique peptides with an FDR of less than 1, S1 and S2 Tables), and 3473 of those were quantified (S3 Table). Of these quantified proteins, 38.57% (1347) proteins were inferred from one peptide and 42.81% (1495) from proteins with more than three unique peptides (Fig 2). The molecular weight and the predicted p*I* of the identified proteins were in the ranges of 2.9–3663.9 kDa and 3.97–12.26, respectively. The mass spectrometry proteomics data have been deposited with the Proteome Xchange Consortium via the PRIDE partner repository with the dataset identifier PXD004472 (username: reviewer36937@ebi.ac.uk; password: 0hqvlzLm).

### Differentially expressed proteins between pre-laying period and laying period

To identify differentially expressed proteins (DEPs) between the pre-laying period and laying period, the ratios of the iTRAQ reporter ions between the pre-laying and laying samples were determined. Student’s *t*-test and the FDR value corresponding to each *p*-value of DEPs were computed. The DEPs were selected according to the following criteria: FDR < 0.05 and iTRAQ ratio > 1.2 or < 0.83. As listed in S4 Table, 684 proteins (418 up-regulated and 266 down-regulated) were extracted.

### Bioinformatics analysis of differentially expressed proteins

**Fig 3:**
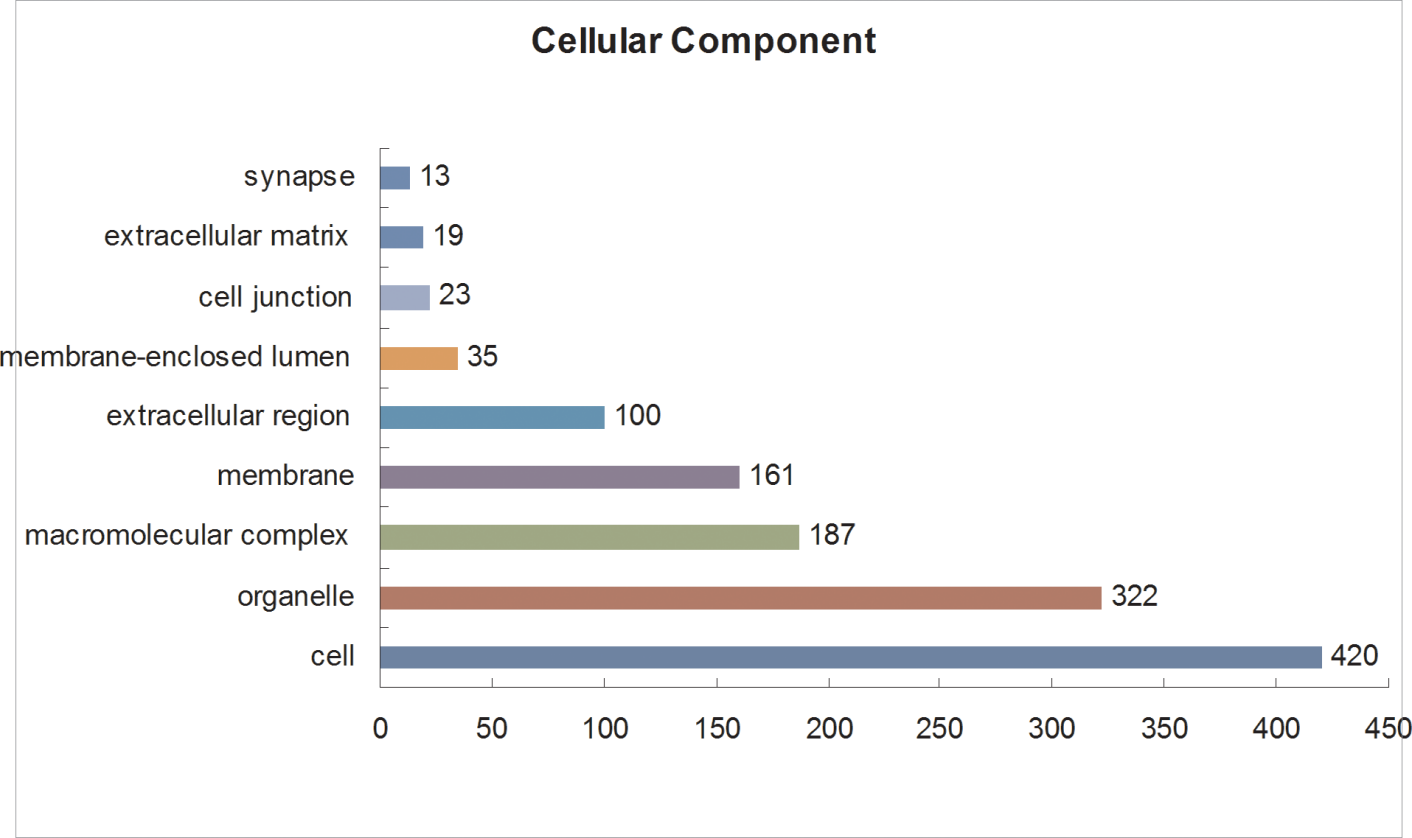
Gene Ontology (GO) cellular component analysis of the differentially expressed proteins in the pituitary gland. All data are presented on the basis of GO second-level terms. Numbers refer to assigned proteins in each category.

**Fig 4:**
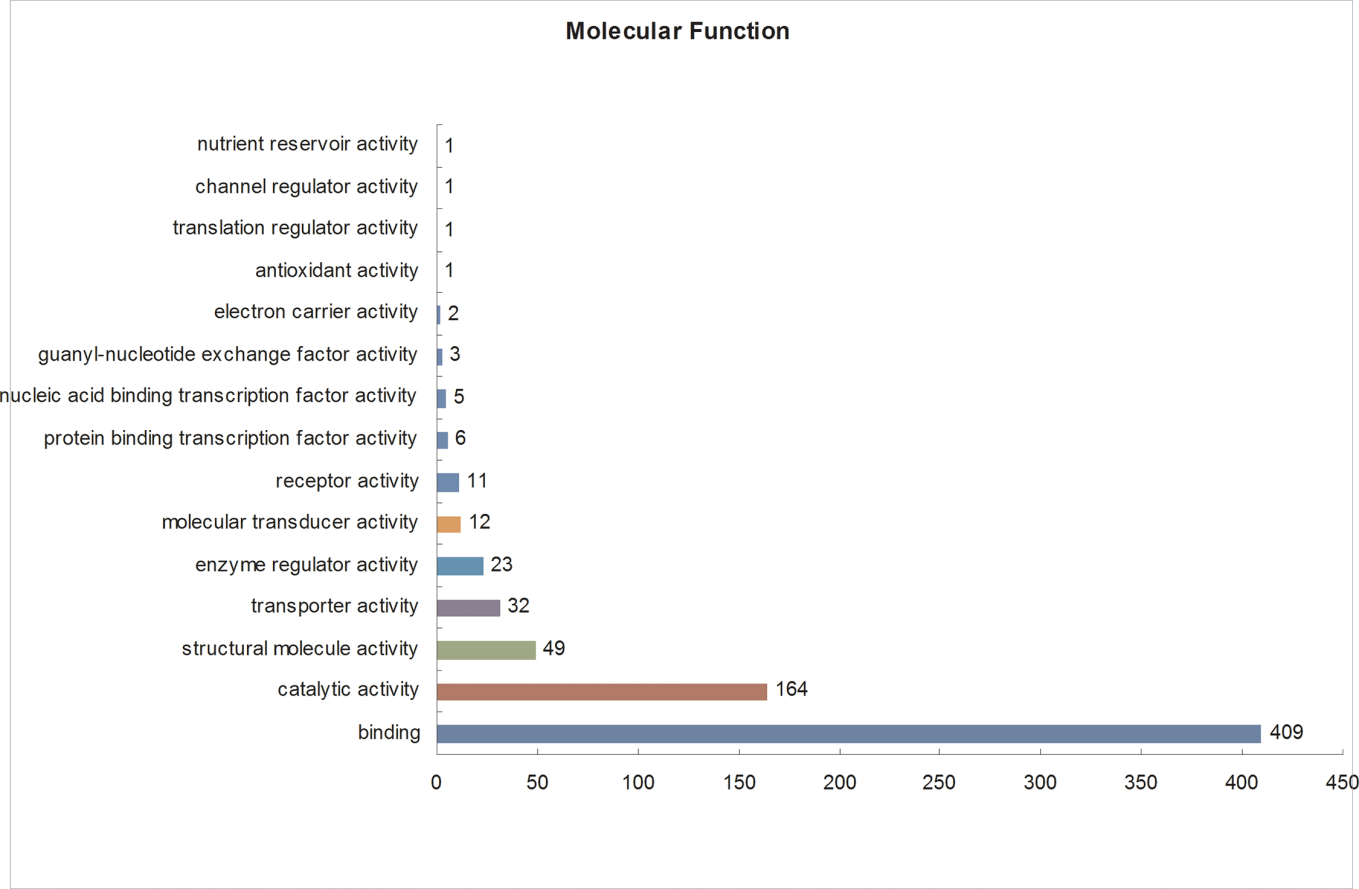
Gene Ontology (GO) molecular function analysis of the differentially expressed proteins in the pituitary gland. All data are presented on the basis of GO second-level terms. Numbers refer to assigned proteins in each category.

**Fig 5:**
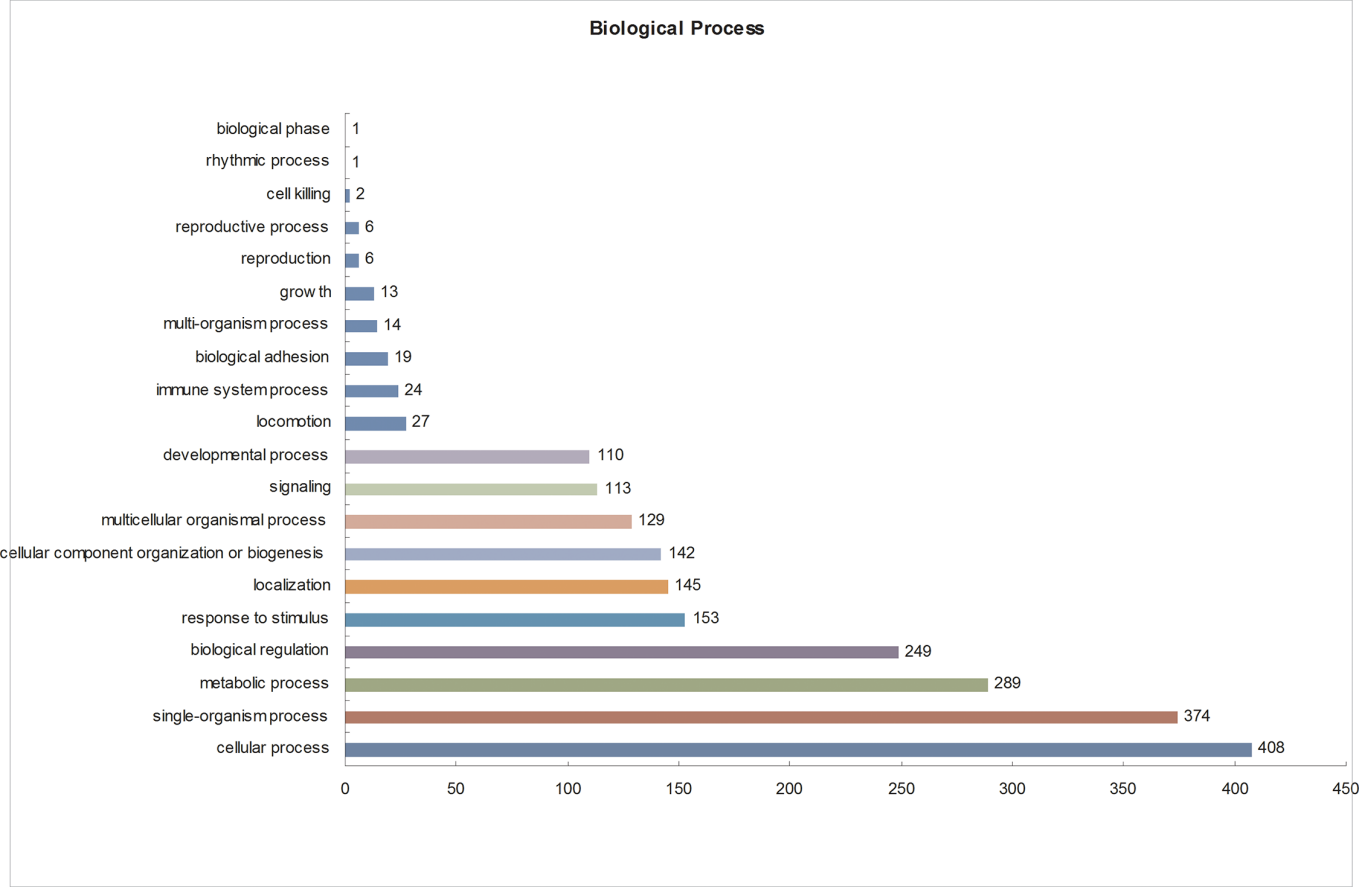
Gene Ontology (GO) biological process analysis of the differentially expressed proteins in the pituitary gland. All data are presented on the basis of GO second-level terms. Numbers refer to assigned proteins in each category.

The Gene Ontology (GO) database is an internationally standardized gene functional classification system that comprehensively describes characteristics of genes and their products. To better understand the differentially expressed proteins, Gene Ontology (GO) annotation was conducted using all 684 identified DEPs. A total of 653 DEPs were annotated to 3820 GO function entries. Second-level GO terms were applied to classify proteins in terms of their involvement in three main categories (cellular component, biological process, and molecular function), and each protein was assigned at least one term. As summarized in S5 Table, in the cellular component category (Fig 3), the differentially expressed proteins were mainly distributed in the cell (420 members), organelles (322 members), macromolecular complex (187 members), membrane (161 members), and extracellular region (100 members), including two secretory granule proteins chromogranin-A (CgA) and secretogranin-2 (SgII). In the molecular function category (Fig 4), the differentially expressed proteins were mostly related to binding (409 members), catalytic activity (164 members), structural molecular activity (49 members), transporter activity (32 members), enzyme regulator activity (23 members), molecular transducer activity (12 members) and receptor activity (11 members). In the biological process category (Fig 5), the differentially expressed proteins were mainly associated with cellular (408 members), single-organism (374 members), and metabolic processes (289 members), as well as biological regulation (249 members). According to these GO classification, some proteins, such as double C2-like domain-containing protein beta, neurophysin 1 and neurophysin 2, were shown to be involved in several important biological processes such as reproduction, hormone secretion and transport, neurotransmitter secretion and transport, and neuropeptide signalling pathways (Table 1). In particular, the abundance of these proteins was increased during the egg-laying period compared with the pre-laying period. Furthermore, some proteins are involved in multiple biological processes, such as Double C2-like domain-containing protein beta, which is involved in calcium ion-dependent exocytosis of neurotransmitters, hormone secretion, hormone transport, neurotransmitter secretion, neurotransmitter transport, regulation of hormone levels, regulation of neurotransmitter levels, and neurophysin 1 and neurophysin 2, which are related to the G-protein coupled receptor signalling and neuropeptide signalling pathways).

**Table 1:** A list of some interesting differentially expressed proteins that are involved in important GO biological processes associated with egg-laying regulation.

GO biological process (Go ID)	Associated Proteins	Protein ID (UniProt Accession)	Gene symbol	Fold change (laying/pre-laying)
**Upregulated proteins**				
calcium ion-dependent exocytosis of neurotransmitter (GO:0048791)	Double C2-like domain-containing protein beta	R0M2S9		2.12
dopamine receptor signalling pathway (GO:0007212)	Guanine nucleotide-binding protein G(Olf) subunit alpha	R0K7A7	GNAL	1.27
female gamete generation (GO:0007292)	Protein diaphanous-like protein 2	R0LBE3	DIAPH2	1.75
glutamate biosynthetic process (GO:0006537)	Delta-1-pyrroline-5-carboxylate synthetase	R0L6Q1		1.55
	Delta-1-pyrroline-5-carboxylate dehydrogenase, mitochondrial	R0K4R7		1.39
glutamate metabolic process (GO:0006536)	Delta-1-pyrroline-5-carboxylate synthetase	R0L6Q1		1.55
	Delta-1-pyrroline-5-carboxylate dehydrogenase, mitochondrial	R0K4R7		1.39
G-protein coupled receptor signalling pathway (GO:0007186)	Neurophysin 1	P35519		2.94
	Neurophysin 2	P19630		2.71
	Uncharacterized protein	U3IJ82	KCTD16	1.75
	Secreted frizzled-related protein 2	R0L3X7	SFRP2	1.57
	Proenkephalin A	R0LY76	PENK	1.45
	Guanine nucleotide-binding protein G(Olf) subunit alpha	R0K7A7	GNAL	1.27
	Rho guanine nucleotide exchange factor 12	R0LEB1		1.25
hormone metabolic process (GO:0042445)	Aldehyde dehydrogenase family 1 member A3	R0LP44		1.26
hormone secretion (GO:0046879)	Double C2-like domain-containing protein beta	R0M2S9		2.12
	Serine/threonine-protein phosphatase 2B catalytic subunit alpha isoform	R0LLX0		1.27
hormone transport (GO:0009914)	Double C2-like domain-containing protein beta	R0M2S9		2.12
	Serine/threonine-protein phosphatase 2B catalytic subunit alpha isoform	R0LLX0		1.27
hormone-mediated signalling pathway (GO:0009755)	Progesterone receptor	R0KDB7		1.48
	Uncharacterized protein	U3J0G8	RXRA	1.39
intracellular steroid hormone receptor signalling pathway (GO:0030518)	Putative ATP-dependent RNA helicase DDX5	R0KA06		1.33
	Protein DJ-1	R0KXC4	PARK7	1.27
neuropeptide signalling pathway (GO:0007218)	Neurophysin 1	P35519		2.94
	Neurophysin 2	P19630		2.71
	Proenkephalin A	R0LY76	PENK	1.45
neurotransmitter secretion (GO:0007269)	Double C2-like domain-containing protein beta	R0M2S9		2.12
	Uncharacterized protein	U3I757	RAB8B	1.48
	Uncharacterized protein	U3IXA7	VAMP3	1.34
	Synaptosomal-associated protein	U3IX10		1.25
neurotransmitter transport (GO:0006836)	Double C2-like domain-containing protein beta	R0M2S9		2.12
	Complexin-1	R0JLF5		1.69
	Uncharacterized protein	U3I757	RAB8B	1.48
	Uncharacterized protein	U3IPK7	SNCA	1.44
	Transporter	U3J6E5	SLC6A17	1.37
	Uncharacterized protein	U3IXA7	VAMP3	1.34
	Protein DJ-1	R0KXC4	PARK7	1.27
	Synaptosomal-associated protein	U3IX10		1.26
neurotransmitter uptake (GO:0001504)	Protein DJ-1	R0KXC4	PARK7	1.27
regulation of androgen receptor signalling pathway (GO:0060765)	Protein DJ-1	R0KXC4	PARK7	1.27
regulation of hormone levels (GO:0010817)	Follicle stimulating hormone alpha polypeptide	B2YI99		3.33
	Double C2-like domain-containing protein beta	R0M2S9		2.12
	Serine/threonine-protein phosphatase 2B catalytic subunit alpha isoform	R0LLX0		1.27
	Aldehyde dehydrogenase family 1 member A3	R0LP44		1.26
regulation of neurotransmitter levels (GO:0001505)	Double C2-like domain-containing protein beta	R0M2S9		2.12
	Uncharacterized protein	U3I757	RAB8B	1.48
	Uncharacterized protein	U3IXA7	VAMP3	1.34
	Protein DJ-1	R0KXC4	PARK7	1.27
	Synaptosomal-associated protein	U3IX10		1.25
Reproduction (GO:0000003)	Uncharacterized protein	U3J3D1	ERCC4	2.14
	Protein diaphanous-like protein 2	R0LBE3	DIAPH2	1.75
	Calmegin	R0L0W9	CLGN	1.72
reproductive process (GO:0022414)	Protein diaphanous-like protein 2	R0LBE3		1.75
	Calmegin	R0L0W9	CLGN	1.72
	Secreted frizzled-related protein 2	R0L3X7	SFRP2	1.57
steroid biosynthetic process (GO:0006694)	Delta-1-pyrroline-5-carboxylate dehydrogenase, mitochondrial	R0K4R7		1.39
	Aldehyde dehydrogenase family 1 member A3	R0LP44		1.26
	Farnesyl pyrophosphate synthetase	R0JP63		1.24
steroid hormone mediated signalling pathway (GO:0043401)	Progesterone receptor	R0KDB7		1.48
	Uncharacterized protein	U3J0G8	RXRA	1.39
steroid metabolic process (GO:0008202)	Apolipoprotein AI	Q9PRR6		1.66
	Delta-1-pyrroline-5-carboxylate dehydrogenase, mitochondrial	R0K4R7		1.39
	Aldehyde dehydrogenase family 1 member A3	R0LP44		1.26
	Farnesyl pyrophosphate synthetase	R0JP63		1.24
Wnt signalling pathway (GO:0016055)	Secreted frizzled-related protein 2	R0L3X7	SFRP2	1.57
	Protein Wnt	R0L9A7		1.29
**Downregulated proteins**				
glutamate metabolic process (GO:0006536)	Excitatory amino acid transporter 1	R0L916		0.79
glutamate receptor signalling pathway (GO:0007215)	Putative glutamate receptor	R0KB27		0.62
G-protein coupled receptor signalling pathway (GO:0007186)	Guanine nucleotide-binding protein G(O) subunit alpha	R0LTA0		0.81
	Diacylglycerol kinase	R0K8R4	DGKE	0.81
	14-3-3 protein gamma	R0KC15		0.80
	Uncharacterized protein	U3I090	KCTD12	0.80
	Uncharacterized protein	U3IMY4	GNAZ	0.71
	Glucagon family neuropeptides	R0LCV3		0.64
hormone secretion (GO:0046879)	Tyrosine-protein phosphatase non-receptor type 11	R0LLA0		0.82
hormone transport (GO:0009914)	Tyrosine-protein phosphatase non-receptor type 11	R0LLA0		0.82
intracellular estrogen receptor signalling pathway (GO:0030520)	Histone-arginine methyltransferase CARM1	R0J6T6		0.72
	Uncharacterized protein	U3IDL9	UFM1	0.71
intracellular steroid hormone receptor signalling pathway (GO:0030518)	Histone-arginine methyltransferase CARM1	R0J6T6		0.72
	Uncharacterized protein	U3IDL9	UFM1	0.71
	Calreticulin	R0KUV7		0.67
neuropeptide signalling pathway (GO:0007218)	Glucagon family neuropeptides	R0LCV3		0.64
neurotransmitter secretion (GO:0007269)	Synaptotagmin-12	R0KKA7		0.83
	Uncharacterized protein	U3INR8	SYN1	0.80
neurotransmitter transport (GO:0006836)	Synaptotagmin-12	R0KKA7		0.82
	Uncharacterized protein	U3INR8	SYN1	0.80
regulation of hormone levels (GO:0010817)	Tyrosine-protein phosphatase non-receptor type 11	R0LLA0		0.82
	Uncharacterized protein	U3HZS2		0.70
regulation of neurotransmitter levels (GO:0001505)	Synaptotagmin-12	R0KKA7		0.82
	Uncharacterized protein	U3INR8	SYN1	0.80
reproduction (GO:0000003)	Heat shock 70 kDa protein	R0LC19		0.82
	Histone H3.3	R0LH29		0.71
	Calreticulin	R0KUV7		0.67
reproductive process (GO:0022414)	Transmembrane emp24 domain-containing protein 2	R0LTH4		0.81
	Uncharacterized protein	U3IHN6	BIRC6	0.54
	Stimulated by retinoic acid gene 6 protein-like protein	R0JRH5	STRA6	0.54
steroid metabolic process (GO:0008202)	Sterol O-acyltransferase 1	R0JWB8		0.67
Wnt signalling pathway (GO:0016055)	Serine/threonine-protein kinase 4	R0LPH1	STK4	0.70

**Fig 6:**
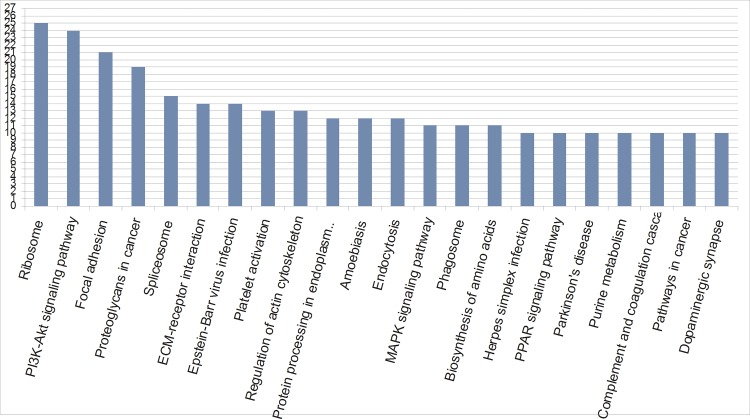
Distribution of KEGG pathways in the pituitary proteome.

Because different proteins interact and cooperate to complete biochemical reactions, following annotation and annotation augmentation, a KEGG pathway-based analysis was performed to identify pathways that would be potentially affected by the modification of the abundance of proteins in the pituitary gland. A total of 328 DEPs were mapped to 251 KEGG pathway entries. All KEGG analysis results are shown in Fig 6 and S6 Table. The KEGG pathway analysis for the identified proteins showed that the top three pathways identified were the ribosome (25 members), PI3K-Akt signalling pathways (24 members), and focal adhesion (21 members). Interestingly, some proteins were mapped for pathways involved in GnRH signalling (ko04912), oocyte meiosis (ko04114), ovarian steroidogenesis (ko04913), oxytocin signalling pathway (ko04921), progesterone-mediated oocyte maturation (ko04914), prolactin signalling pathway (ko04917), and estrogen signalling pathway (ko04915). These proteins included follicle stimulating hormone alpha polypeptide, mitogen-activated protein kinase kinase kinase 2, ITPR3, prolactin, progesterone receptor, neurophysin 1, and guanine nucleotide-binding protein G(O) subunit alpha (Table 2, S6 Table and S1 Fig).

**Table 2:** A list of some interesting differentially expressed proteins that are involved in important pathways associated with egg-laying regulation.

Pathway (Map ID)	Associated Proteins	Protein ID (UniProt Accession)	Gene symbol	Fold change (laying/pre-laying)
**Upregulated proteins**				
Calcium signalling pathway (ko04020)	Uncharacterized protein	U3IUD9	RYR2	3.61
	Myosin light chain kinase, smooth muscle	R0LM85	MYLK	1.38
	Serine/threonine-protein phosphatase	R0L7Z2		1.28
	Guanine nucleotide-binding protein G(Olf) subunit alpha	R0K7A7	GNAL	1.27
Dopaminergic synapse (ko04728)	Serine/threonine-protein phosphatase	R0L7Z2		1.28
	Guanine nucleotide-binding protein G(Olf) subunit alpha	R0K7A7	GNAL	1.27
GABAergic synapse (ko04727)	Uncharacterized protein	U3J3R3		1.39
	Vesicular inhibitory amino acid transporter	R0LZ13		1.37
Glutamatergic synapse (ko04724)	Uncharacterized protein	U3J3R3		1.39
	Serine/threonine-protein phosphatase	R0L7Z2		1.28
GnRH signalling pathway (ko04912)	Follicle stimulating hormone alpha polypeptide	B2YI99		3.33
	Mitogen-activated protein kinase kinase kinase 2	R0KWS2	MAP3K2	1.86
MAPK signalling pathway (ko04010)	Uncharacterized protein	U3I9X9		2.20
	Mitogen-activated protein kinase kinase kinase 2	R0KWS2		1.86
	Stathmin	R0LGK3		1.66
	Microtubule-associated protein	U3IKL9	MAPT	1.52
	Uncharacterized protein	U3IDW4	ARRB1	1.31
	Serine/threonine-protein phosphatase	R0L7Z2		1.28
	Uncharacterized protein	U3IPY8	FLNB	1.21
Oocyte meiosis (ko04114)	Progesterone receptor	R0KDB7		1.48
	Serine/threonine-protein phosphatase	R0L7Z2		1.28
Ovarian steroidogenesis (ko04913)	Follicle stimulating hormone alpha polypeptide	B2YI99		3.33
Oxytocin signalling pathway (ko04921)	Uncharacterized protein	U3IUD9	RYR2	3.61
	Neurophysin 1	P35519		2.94
	Myosin light chain kinase, smooth muscle	R0LM85	MYLK	1.38
	Serine/threonine-protein phosphatase	R0L7Z2		1.28
	Uncharacterized protein	U3IVI4	PPP1R12B	1.21
Progesterone-mediated oocyte maturation (ko04914)	Progesterone receptor	R0KDB7		1.48
Prolactin signalling pathway (ko04917)	Follicle stimulating hormone alpha polypeptide	B2YI99		3.33
Steroid biosynthesis (ko00100)	Bile salt-activated lipase	R0LW36		1.54
Wnt signalling pathway (ko04310)	Secreted frizzled-related protein 2	R0L3X7	SFRP2	1.88
	Protein Wnt	R0L9A7		1.29
	Serine/threonine-protein phosphatase	R0L7Z2		1.28
	Uncharacterized protein	U3IX89	DAAM2	1.21
**Downregulated proteins**				
Calcium signalling pathway (ko04020)	Uncharacterized protein	U3I675	ITPR3	0.80
	Uncharacterized protein	U3J4U9	CAMK2A	0.62
	7.8S IgY heavy chain	A0A024B6A7		0.61
Dopaminergic synapse (ko04728)	Amine oxidase	R0M1F3		0.82
	Guanine nucleotide-binding protein G(O) subunit alpha	R0LTA0		0.81
	Uncharacterized protein	U3I675	ITPR3	0.80
	Serine/threonine-protein phosphatase 2A 56 kDa regulatory subunit delta isoform	R0KZ66		0.78
	Serine/threonine-protein phosphatase 2A 56 kDa regulatory subunit alpha isoform	R0LRK2		0.73
	Tyrosine 3-monooxygenase	R0JUR5	TH	0.71
	Uncharacterized protein	U3J4U9	CAMK2A	0.62
	Uncharacterized protein	U3I684	DDC	0.52
Estrogen signalling pathway (ko04915)	Heat shock 70 kDa protein	R0LC19	HSP70	0.82
	Guanine nucleotide-binding protein G(O) subunit alpha	R0LTA0		0.81
	Uncharacterized protein	U3I675	ITPR3	0.80
GABAergic synapse (ko04727)	Guanine nucleotide-binding protein G(O) subunit alpha	R0LTA0		0.81
	Uncharacterized protein	U3IHF0	GAD2	0.53
Glutamatergic synapse (ko04724)	Guanine nucleotide-binding protein G(O) subunit alpha	R0LTA0		0.81
	Uncharacterized protein	U3I675	ITPR3	0.80
	Excitatory amino acid transporter 1	R0L916		0.79
GnRH signalling pathway (ko04912)	Uncharacterized protein	U3I675	ITPR3	0.80
	Uncharacterized protein	U3J4U9	CAMK2A	0.62
MAPK signalling pathway (ko04010)	Heat shock 70 kDa protein	R0LC19	HSP70	0.82
	Uncharacterized protein	U3I603	CACNA2D1	0.76
	Serine/threonine-protein kinase 4	R0LPH1	STK4	0.70
	Tyrosine-protein phosphatase non-receptor type 5	R0KPE1		0.64
Oocyte meiosis (ko04114)	14-3-3 protein gamma	R0KC15		0.80
	14-3-3 protein theta	R0LY14		0.80
	Uncharacterized protein	U3I675	ITPR3	0.80
	Serine/threonine-protein phosphatase 2A 56 kDa regulatory subunit delta isoform	R0KZ66		0.78
	Serine/threonine-protein phosphatase 2A 56 kDa regulatory subunit alpha isoform	R0LRK2		0.73
	Uncharacterized protein	U3J4U9	CAMK2A	0.62
Oxytocin signalling pathway (ko04921)	Guanine nucleotide-binding protein G(O) subunit alpha	R0LTA0		0.81
	Uncharacterized protein	U3I675	ITPR3	0.80
	Uncharacterized protein	U3I603	CACNA2D1	0.76
	Uncharacterized protein	U3J4U9	CAMK2A	0.62
Progesterone-mediated oocyte maturation (ko04914)	Mitotic spindle assembly checkpoint protein MAD1	R0L5S6		0.64
Prolactin signalling pathway (ko04917)	Prolactin	R0K3E3		0.79
	Prolactin	Q4PLT4		0.74
	Tyrosine 3-monooxygenase	R0JUR5	TH	0.71
Serotonergic synapse (ko04726)	Amine oxidase	R0M1F3		0.82
	Guanine nucleotide-binding protein G(O) subunit alpha	R0LTA0		0.81
	Uncharacterized protein	U3I675	ITPR3	0.80
	Uncharacterized protein	U3I684	DDC	0.52
Steroid biosynthesis (ko00100)	Sterol O-acyltransferase 1	R0JWB8		0.67
Wnt signalling pathway (ko04310)	Uncharacterized protein	U3J9H3	CSNK2A2	0.81
	Casein kinase II subunit alpha	R0JGR8		0.81
	Uncharacterized protein	U3J4U9	CAMK2A	0.62
	Vang-like protein 1	R0LBD3		0.31

### Western blot validation

**Fig 7:**
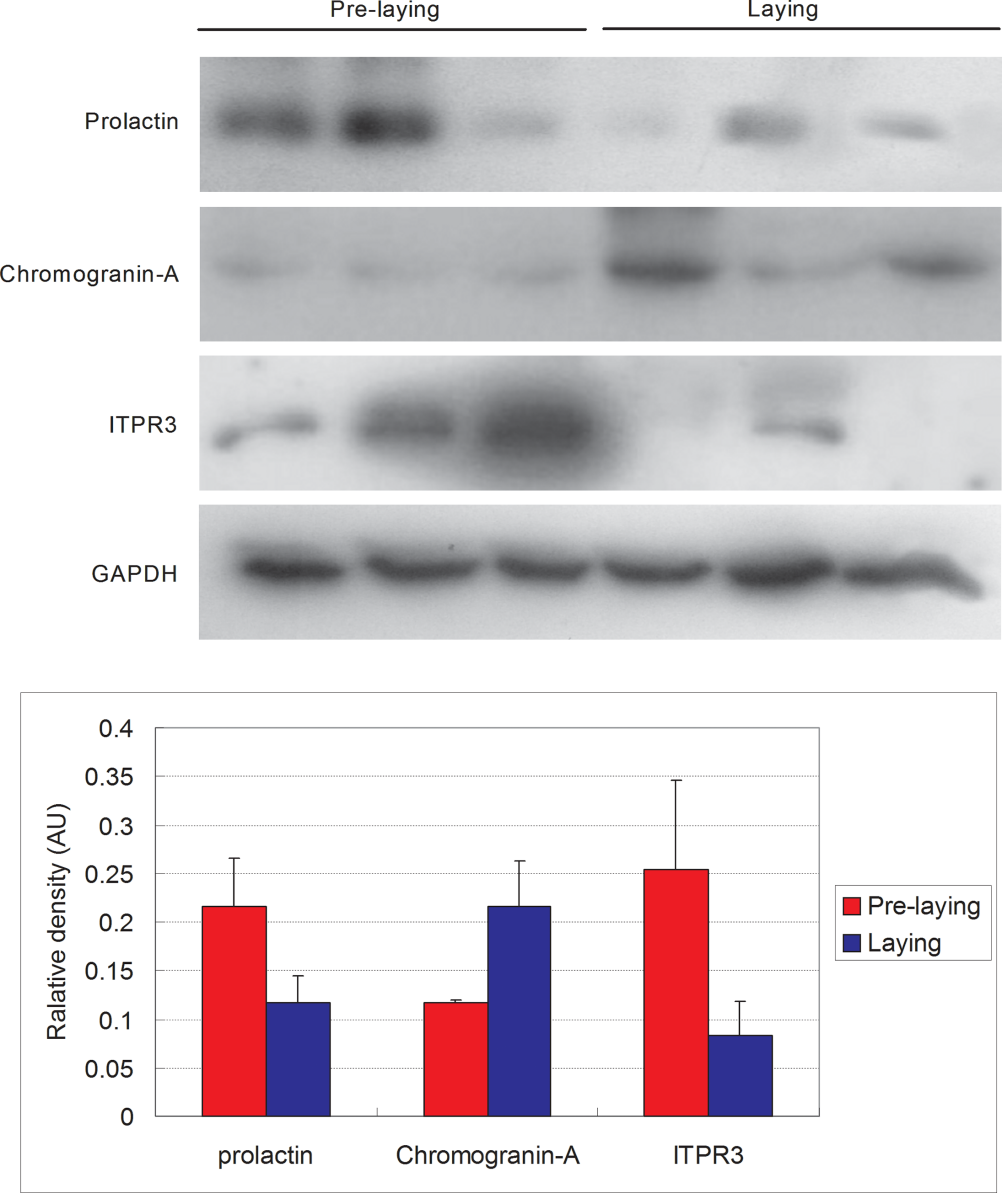
Western blot analysis of prolactin, chromogranin-A and ITPR3 in the pituitary tissues of Huoyan geese between pre-laying and laying periods. GAPDH was used as the internal control. Protein band density was analysed using the GelQuant software. Upper panels: representative immunoblots. Lower panels: densitometric analysis of prolactin, chromogranin-A and ITPR3 protein relative to GAPDH protein. Values are expressed as the means±SEM of the mean in arbitrary optical density units (AU).

To further validate results obtained by iTRAQ labelling and LC-MS/MS, we examined the modification of the abundance of proteins such as prolactin, chromogranin-A and ITPR3 in the pre-laying and laying period pituitary tissues using traditional western blotting. As shown in Fig 7, the abundance of chromogranin-A was increased in the laying period compared with the pre-laying period (0.2167±0.0463 vs 0.1167±0.0033). The abundance of prolactin was decreased in the laying period compared with the pre-laying period (0.1167±0.0273 vs 0.2167±0.0484). Similarly, the abundance of ITPR3 was also decreased in the laying period compared with the pre-laying period (0.0833±0.0035 vs 0.2533±0.0092). In comparison with the results obtained using proteomics, the changes in the trends in the abundance of these three proteins were consistent with the observations made by iTRAQ, although the difference were not statistically significant (p>0.05).

## Discussion

In this study, we performed a comprehensive evaluation of the proteomic profile in the pituitary gland of Huoyan geese between the pre-laying and laying periods. A total of 684 differentially expressed proteins were identified. After GO annotation, some differentially expressed proteins were found to be associated with hormone secretion and transport, neurotransmitter secretion and transport, regulation of hormone and neurotransmitter levels, G-protein coupled receptor signalling, neuropeptide signalling, reproductive processes, and steroid hormone mediated signalling. Such proteins included double C2-like domain-containing protein beta, neurophysin 1 and 2, follicle stimulating hormone alpha polypeptide, complexin-1, secreted frizzled-related protein 2, and progesterone receptors. By analysing the KEGG pathway of differentially expressed proteins, we found that several proteins, such as follicle stimulating hormone alpha polypeptide, prolactin (PRL), mitogen-activated protein kinase kinase kinase 2, ITPR3, ryanodine receptor 2, progesterone receptor, and neurophysin 1, could be categorized as being involved in GnRH signalling, oocyte meiosis, ovarian steroidogenesis, progesterone-mediated oocyte maturation, prolactin signalling, calcium signalling, oxytocin signalling, oestrogen signalling, and glutamatergic synapses.

As the basis of egg-laying, ovarian follicular development is subject to regulations by numerous endocrine and autocrine/paracrine factors that include pituitary gonadotropins, growth factors, and PRL. It is well known that the pituitary gland is the source of important physiological hormones. Gonadotropins including follicle-stimulating hormone (FSH) and luteinizing hormone (LH) are synthesized in and secreted from the pituitary gland. These hormones are glycoprotein hormones and are composed of two dissimilar subunits, α and β. In a given species, the α-subunit is identical among these pituitary glycoprotein hormones, whereas the β-subunits are different and determine the hormonal and species specificity [31]. Avian follicular development depends on a complex interaction of complex biochemical and physiological processes. As a reproductive axis hormone, FSH is a key regulator for follicular development and the selection of the dominant follicle for ovulation. It has been reported to be crucial for steroidogenesis, follicular recruitment and growth, and increasing the division rates of the granulosa cell associated with follicular selection in avian ovaries [32–34]. The formation and secretion of FSH are mainly regulated by hypothalamic and gonadal factors, such as GnRH and gonadal steroid hormones. In this study, the abundance of follicle stimulating hormone alpha polypeptide was significantly increased during the laying period. This increase may indicate that the secretion of pituitary gonadotropins was activated to meet the growth and differentiation of the ovaries and ovulation. Furthermore, another hormone secreted by the pituitary gland, PRL, has been reported to be involved in the regulation of many important physiological processes, including egg laying, induction and maintenance of incubation behaviour, osmoregulation, immune modulation, and gonadal development and functions [35, 36]. Notably, PRL is considered a negative regulator of avian reproductive processes. PRL plays an inhibitory role in avian reproductive processes at all levels of the hypothalamic-pituitary-gonadal axis via inhibition of gonadotropin secretion, stimulation of incubation behaviour, and development of atresia in ovarian follicles [37, 38]. Our proteomic analysis showed that the abundance of PRL during the laying period was decreased compared to that of the pre-laying period, this modification might be responsible for reducing the inhibitory effect of PRL on reproductive processes.

In this study, the abundance of two inert carrier proteins for neurohypophysial hormones, neurophysin-1 and neurophysin-2, were found to be increased during the laying period. In mammals, arginine vasopressin and oxytocin are two types of neurohypophysial hormones that are synthesized in the hypothalamus, from where they are axonally transported to the posterior pituitary and presented as non-covalently bound complexes with their designated neurophysin in the secretory granules of the posterior pituitary. Precursors of these neurohypophysial hormones are small proteins processed into nonapeptide hormones and neurophysins during axonal transport to the neurohypophysis. Therefore, the neurophysins are generally considered to be biologically inert carrier proteins for oxytocin and vasopressin. There are two major neurophysins: neurophysin-1 and neurophysin-2. Neurophysin 1 is the carrier protein of oxytocin, and neurophysin 2 is the carrier protein of vasopressin. Because the two neurohypophysial hormones are packaged together with neurophysin in secretory granules, a neurophysin is also released when a neurohypophysial hormone is released [39]. In avian species, vasopressin and oxytocin are replaced by arginine vasotocin (AVT) and mesotocin (MT), respectively, and they have also two types of neurophysins associated with the corresponding precursors [40, 41]. As two important avian neurohypophysial hormones, AVT and MT are synthesized by hypothalamic magnocellular neurons and secreted from the posterior pituitary gland as in mammals. AVT has well-documented oxytocic effects on various reproductive behaviours and stimulatory effects on shell gland contractility and oviposition [42]. Oviposition, which is the expulsion of the egg from the shell gland of the bird oviduct, generally occurs approximately 30 min before the ovulation of the next follicle and is initiated by contractions of the uterine smooth muscles. AVT is known to cause the contractions of the smooth muscles of the uterus in birds through increased binding to its receptor existing in the tissue. The distinct peak of AVT release from the pituitary defines the oviposition cycle for each bird [43]. An injection of AVT can induce premature oviposition in laying hens. Though the synthesis of AVT is also stimulated by oviposition, the AVT level in the plasma, paraventricular nucleus (PVN) and supraoptic nucleus (SON) increases after oviposition [44]. In addition, AVT expression in hypothalamic neurons may depend on the levels of gonadal hormones and other neuroendocrine systems. Testosterone and estradiol have been shown to strongly activate vasotocin mRNA expression in the bed nucleus of the stria terminalis (BSTM) in female Japanese quail [45, 46]. Mesotocin (MT), however, appears not to be involved to a major degree in the physiological functions of neurohypophysial hormones. However, MT has also been demonstrated to enhance the induction of oviposition by AVT through an increase in the sensitivity of the uterus to AVT at oviposition in hens [47]. In our study, the increased abundance of neurophysin-1 and neurophysin-2 during the laying period benefits the secretion and transport of AVT and MT and subsequently promotes ovulation.

In pituitary gland cells, the release of neurotransmitters and hormones is triggered by Ca^2+^ binding to a presynaptic Ca^2+^ sensor that induces synaptic vesicle exocytosis with a high degree of Ca^2+^ cooperativity [48]. In this study, the abundance of double C2-like domain-containing protein beta was found to be significantly increased in the laying period. The C2 domain (calcium/lipid-binding domain) is a Ca^2+^-dependent membrane-targeting module found in many cellular proteins involved in signal transduction or membrane trafficking. C2 domains are unique among membrane-targeting domains in that they show a wide range of lipid selectivity for the major components of cell membranes, including phosphatidylserine and phosphatidylcholine. The C2 domain is thought to be involved in Ca^2+^-dependent phospholipid binding and in membrane-targeting processes such as subcellular localization. To date, more than 40 distinct C2 domains have been identified, for example in proteins such as synaptotagmin, phospholipase A2, GTPase-activating proteins, and phospholipase C isoforms. Synaptotagmin can preferentially undergo distinct modes of exocytosis with different forms of stimulation, which can shape Ca^2+^ sensing in endocrine cells, contributing to the regulation of hormone release and the organization of complex endocrine functions. In our previous study, the mRNA expression of synaptotagmin-1, another protein containing two conserved C2 domains, was over-expressed in the pituitary gland of Huoyan geese in the laying period compared to the ceased period. The conserved C2 domains of synaptotagmin-1 are referred to as the C2A and C2B domains. Both the C2A and the C2B domains bind Ca^2+^; the C2B domain, which exhibits Ca^2+^/phospholipid binding activity, is the major Ca^2+^ sensor for fast synchronous neurotransmitter release, and Ca^2+^ binding to the C2A domain is a major regulator of Ca^2+^ binding to the C2B domain and contributes to the overall Ca^2+^ cooperativity of neurotransmitter release [49, 50]. Both synaptotagmin-1 and double C2-like domain-containing protein may involve the regulation of Ca^2+^-dependent exocytosis. Exocytosis is a key biological process that controls the neurotransmission and release of secretory products from neurons and other secretory cell types. Neurotransmitters, hormones, or other secretory products are packed in vesicles, with a number of these vesicles fusing with the surface membrane during both non-activated and activated phases, to release secretory products into the extracellular space [51].

We found that the abundance of ITPR3, a member of the family of inositol 1,4,5-trisphosphate (IP_3_) receptors, was modified. As noted above, anterior pituitary hormone secretion is a Ca^2+^-dependent process. Inositol 1,4,5-trisphosphate (IP_3_) is an intracellular second messenger that transduces neurotransmitter signals. IP_3_ mediates the release of Ca^2+^ from intracellular stores by binding to its specific Ca^2+^ channel-coupled receptors [52]. Thus, IP_3_ receptors play a pivotal role in linking G-protein-coupled receptor (GPCR)-mediated IP_3_ formation to increases in cytoplasmic free Ca^2+^ concentration [53]. GnRH acts on pituitary gonadotropes via a G-protein coupled receptor to increase cytosolic Ca^2+^ and stimulate exocytotic hormone secretion. During these processes, the binding of GnRH to G protein coupled receptors on the cell membrane of gonadotropic cells triggers a second messenger cascade in which inositol 1,4,5-trisphosphate (IP_3_) is produced. IP_3_ in turn binds to its receptor-activated Ca^2+^ channels on the endoplasmic reticulum (ER). Upon binding, these channels open and calcium flows from the ER into the cytosol, resulting in intracellular calcium oscillations. These oscillations trigger the secretion of gonadotropin secretion [54]. However, sustained exposure to GnRH is able to reduce GnRH-stimulated gonadotropin secretion. GnRH can induce inositol 1,4,5-trisphosphate receptor down-regulation utilizing the ubiquitin/proteasome pathway and contribute to the suppression of LH/FSH secretion [53]. In αT3-1 mouse anterior pituitary gonadotropes, chronic activation of gonadotropin-releasing hormone (GnRH) receptors causes inositol 1,4,5-trisphosphate receptor down-regulation [55]. In our study, the decrease in the abundance of ITPR3 during the laying period may be due to negative feedback from GnRH or LH/FSH.

It is worth mentioning that the abundance of two major granin proteins, secretogranin II (SgII) and chromogranin A (CgA), were shown to be increased during the laying period. The “granin” protein family includes chromogranin A (CgA), chromogranin B (CgB/secretogranin I), and chromogranin C (CgC/secretogranin II). These granins are found in the secretory granules of a broad variety of endocrine and neuroendocrine cells and are released from the cell along with the hormonal component of the granule in response to a stimulus. Granins serve in the sorting and aggregation of secretory products in the trans-Golgi network (TGN) and the subsequent formation of secretory granules [56]. Granins are thought to function in hormone packaging within secretory granules, in hormone secretion, and as pro-hormones for various proteolytic cleavage products [57]. Normal reproductive function is dependent on the production of gonadotropins (LH and FSH) from specialised gonadotroph cells in the anterior pituitary. Synthesis and secretion of gonadotropins are regulated by pulsatile release of GnRH from the hypothalamus and by the negative feedback effects of gonadal steroids, acting either directly at the pituitary or indirectly by regulating GnRH release from the hypothalamus [58, 59]. Several studies have demonstrated the co-localization and co-aggregation of secretogranin-II (SgII) with luteinizing hormone (LH) in secretory vesicles of gonadotrophs and the co-release of these hormones in response to various GnRH regimes. Using the model of LβT2 mouse gonadotroph cells, Nicol et al. reported that there is a close correlation between the GnRH-stimulated release of LH and SgII [60, 61]. In the pituitary gland of GnRH-deprived male mice, there is preferential storage of SgII and a subsequent increase in intragranular coaggregation with LH [62]. CgA plays a major role in the constitutive secretion of FSH. There is evidence to suggest that CgA and FSH are found within the same type of granule within gonadotrophs [63], and FSH is packaged with CgA in male rat gonadotrophs [64]. These results suggest that the granin proteins SgII and CgA may have a regulatory role involving differential secretion of gonadotropins such as LH and/or FSH. Similar to our results, for avian species, CgA has been revealed to be present in bird gonadotrophs and to modulate hormone secretion [56, 65]. In the hypothalamus and pituitary gland of high-egg-production chicken strains, the mRNA expression levels of SgII are significantly higher than in low-egg-production strains [5].

It needs to be explained that the sequence database searching in this study was mapped to the uniprot_anatidae_34815_20140815. Even the comparative genomic analysis revealed that geese and ducks were most likely derived from a common ancestor approximately 20.8 million years ago, and the goose genome has a high synteny with the duck genome [66], but which covered approximately 81.09% and 82.35% of each genome, respectively [67]. Therefore, it is best to compare the proteins sequences with the data from the genome project for the swan goose. Furthermore, Genomewide comparisons and orthologous analyses will be useful to determine special characteristics of geese and facilitate future genetic breeding programs. At present, the goose genome sequences have been published [67, 68], it makes possible to compare obtained proteins sequences with the data from these genome project for the swan goose in subsequent study.

## Conclusions

In summary, our study is the first to screen the differentially expressed proteins of the pituitary gland of Huoyan geese during the laying period compared to the pre-laying period using proteomics technology. Several proteins were identified, including double C2-like domain-containing protein, neurophysin 1 and 2, secretogranin II, chromogranin A, prolactin, ITPR3, and follicle stimulating hormone alpha polypeptide, and they were related to the regulation of secretion and transport of hormones and neurotransmitters and to the oxytocin signalling, GnRH signalling, prolactin signalling, and calcium signalling pathways. Our findings may provide important information for the conservation and utilization of local goose breeds.

## Supporting information

S1 Table All identified proteins in the pituitary between the pre-laying period and laying period by iTRAQ analysis. (XLS)

S2 Table Raw data of peptide quantification of the pituitary between the pre-laying period and laying period. (XLS)

S3 Table Significance analysis of all quantified proteins in the pituitary between the pre-laying period and laying period. (XLS)

S4 Table Significant differentially expressed proteins (DEPs) in the pituitary between the pre-laying period and laying period. (XLS)

S5 Table GO analysis result for differentially expressed proteins (DEPs) in the pituitary. (XLS)

S6 Table KEGG analysis results for differentially expressed proteins (DEPs) in the pituitary. (XLS)

S1 Fig Representative KEGG pathway maps. (DOC)
