# Supplementary figures and images for: Comparative proteomic analysis of pituitary glands from Huoyan geese between pre-laying and laying periods using an iTRAQ-based approach
Source: PLoS One. 2017 Sep 25;12(9):e0185253. doi: 10.1371/journal.pone.0185253 (PMC5612699; doi:10.1371/journal.pone.0185253)

Supplemental figures. Representative KEGG pathway

ko04912


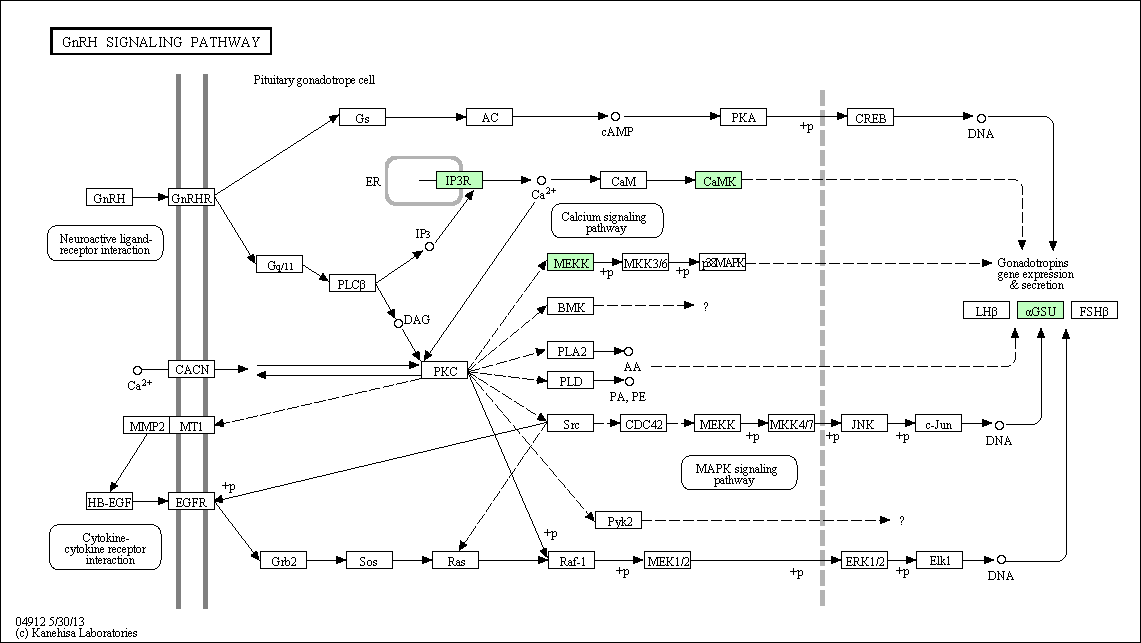


ko04114


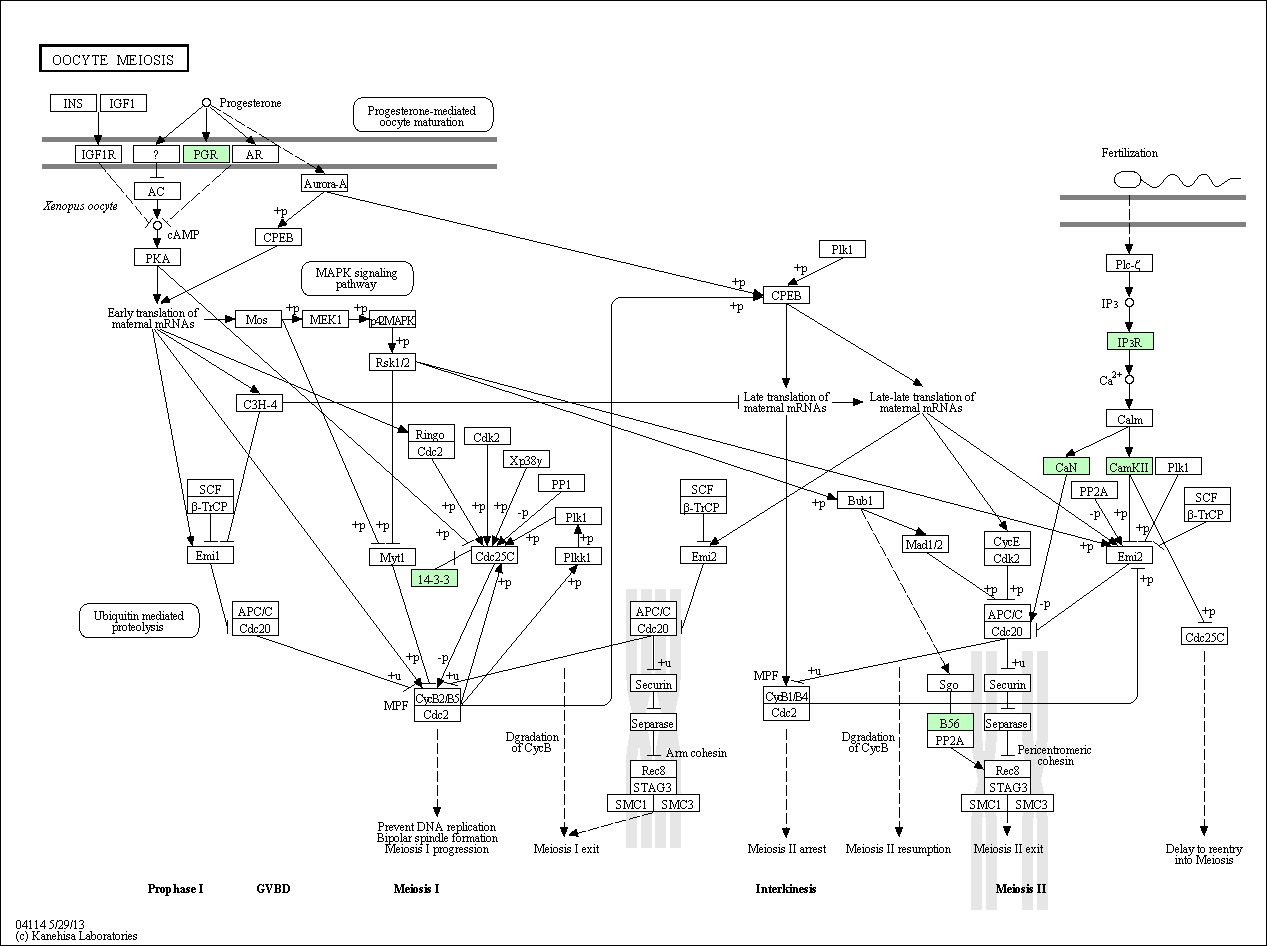


ko04913


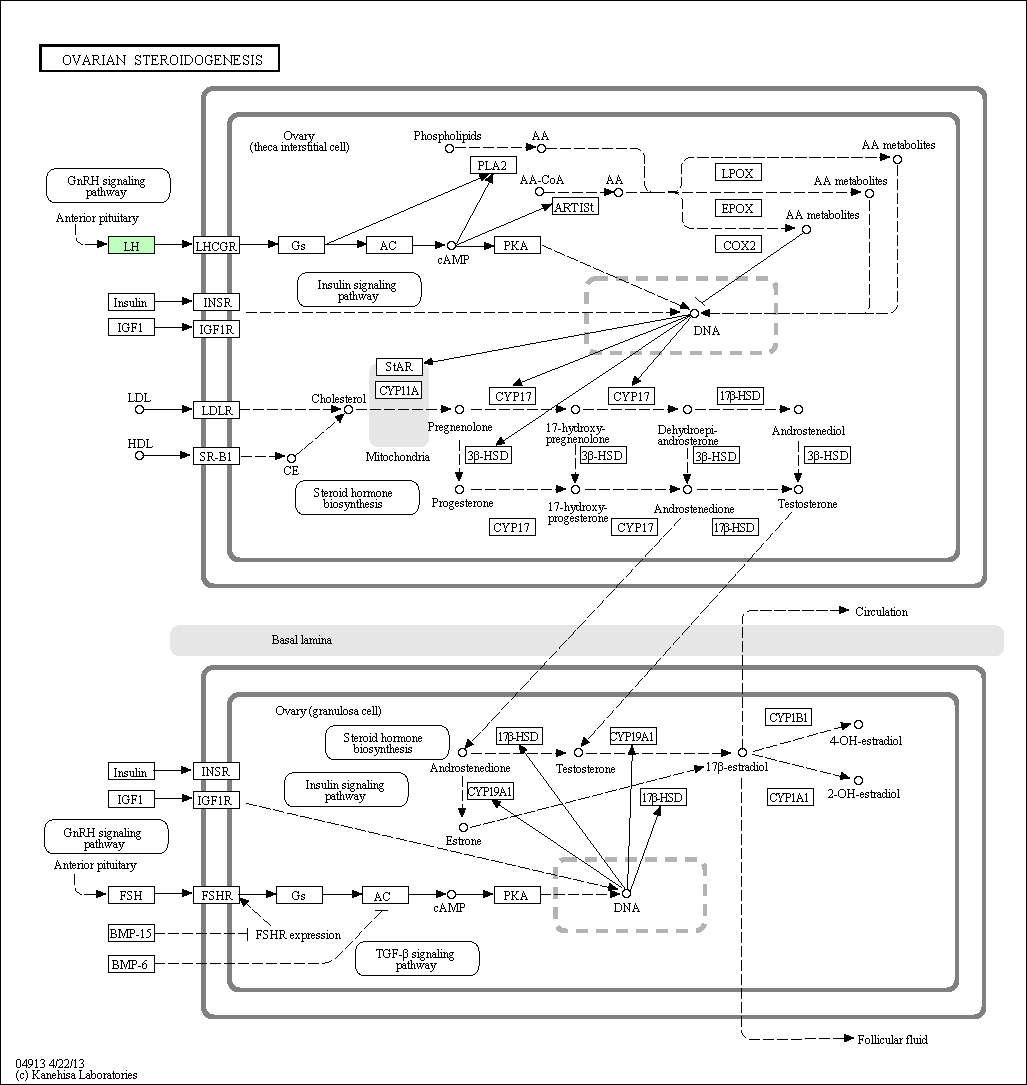


ko04921


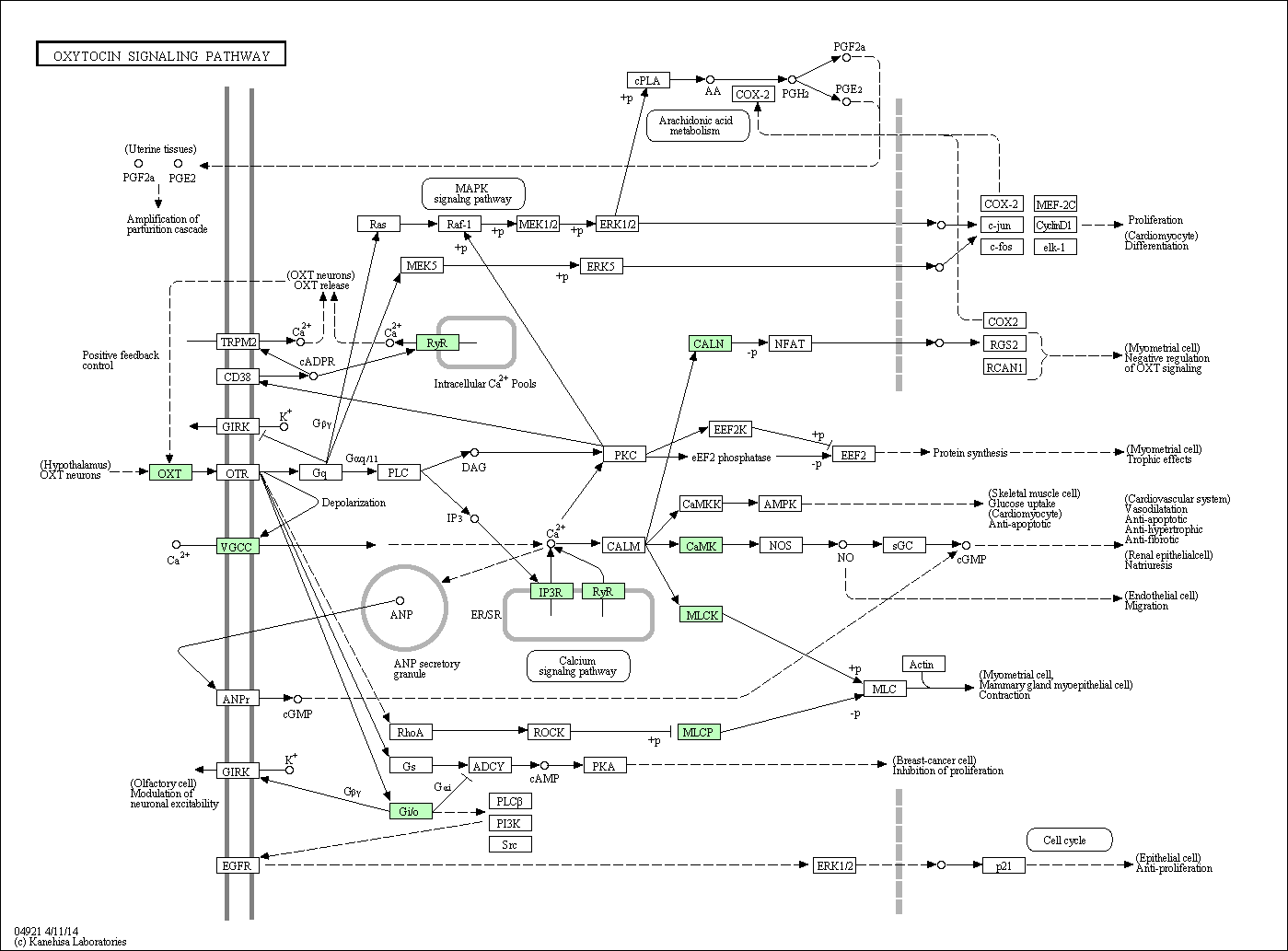


ko04914


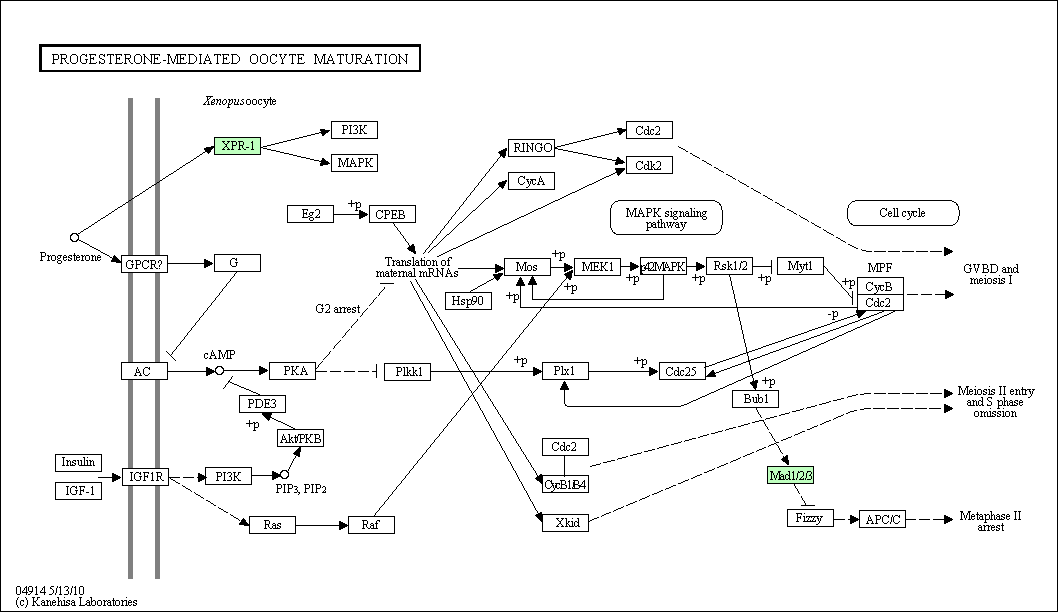


ko04917


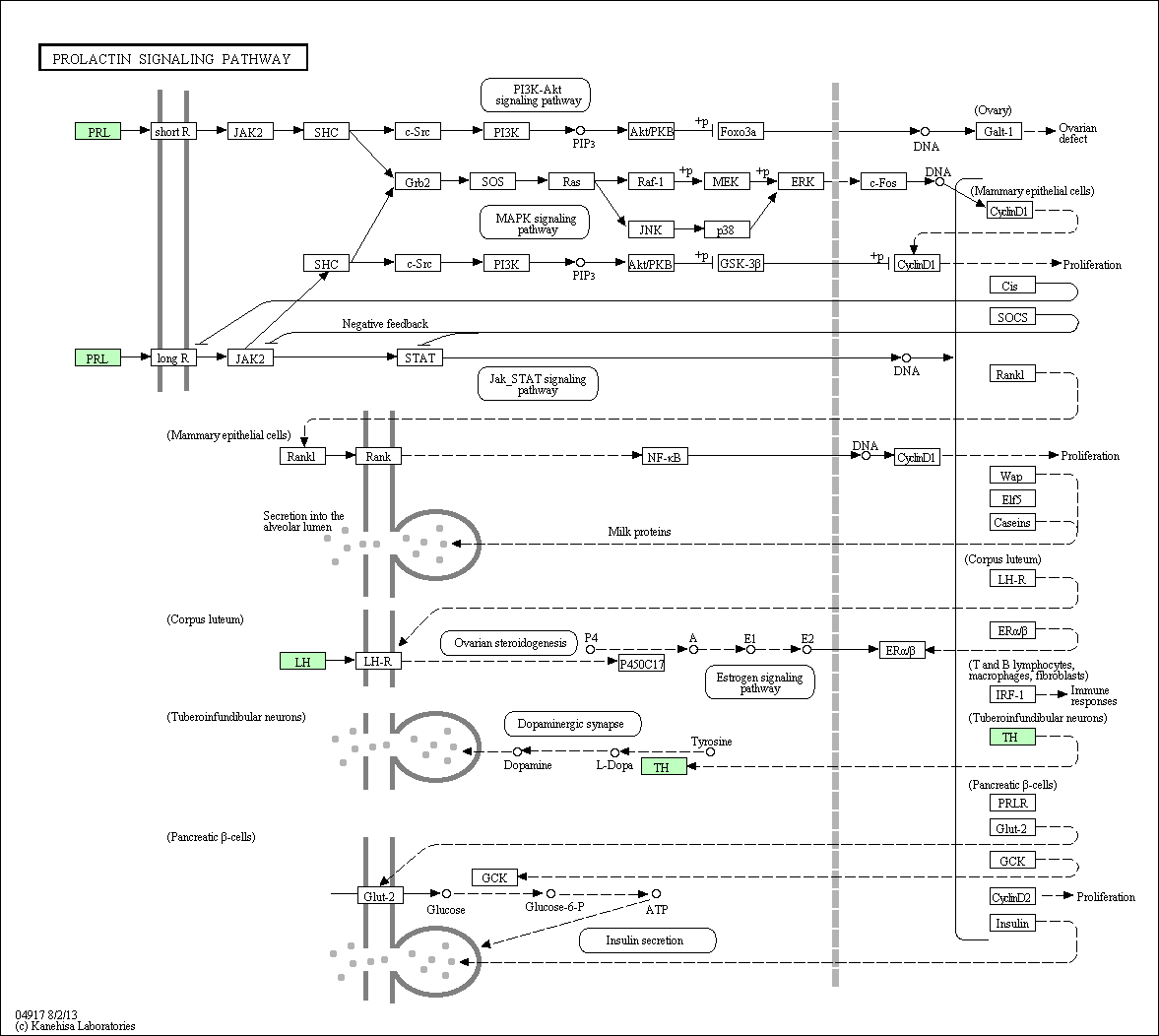


ko04915


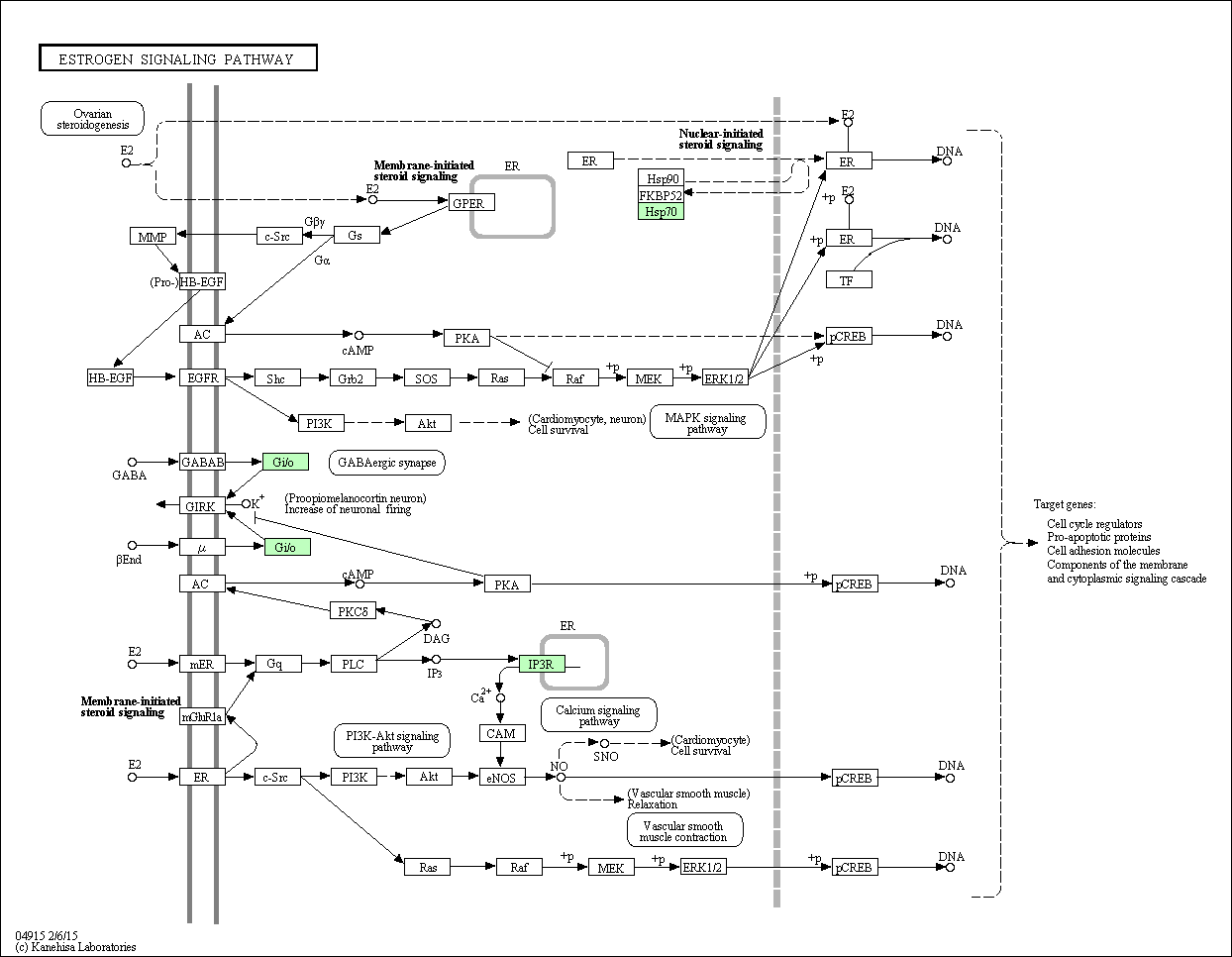

Supplement: S1 Fig — (DOC) [file pone.0185253.s007.doc]
